# Sleep Hours, Wake-Up Time, Bedtime, Breakfast Skipping, and Japanese-Style Diet (Staple Food, Main Dish, and Side Dish): A Cross-Sectional Study of Schoolchildren in Saga Prefecture

**DOI:** 10.3390/children13010030

**Published:** 2025-12-25

**Authors:** Aya Sato, Yuki Sato, Chieko Suzuki, Reiko Suzuki

**Affiliations:** 1 Department of Nutritional Science, Faculty of Food and Nutritional Sciences, Japan Women’s University, Tokyo 112-8681, Japan; m2222301sa@gr.jwu.ac.jp; 2 Division of Medical Nutrition, Faculty of Healthcare, Tokyo Healthcare University, Tokyo 154-8568, Japan; 3 National Institute of Occupational Safety and Health, Tokyo 204-0024, Japan; sato-y@h.jniosh.johas.go.jp; 4 Department of Nursing Faculty of Medicine, Saga University, Saga 849-8501, Japan; chiekosu@cc.saga-u.ac.jp

**Keywords:** sleep hours, wake-up time, bedtime, breakfast skipping, Japanese-style diet, schoolchildren

## Abstract

Objectives: We investigated the association between children’s waking time and bedtime and contents of home meals, focusing on ‘skipping breakfast’ and ‘meal of staple food, main dish, and side dish’ (SMS meal). Methods: A cross-sectional survey concerning children’s lifestyle habits and dietary habits was conducted at seven primary schools within Saga Prefecture in northern Kyushu, Japan, with 2457 parents/guardians participating. Logistic regression analyses were used to estimate odds ratios (ORs) and 95% confidence intervals (CIs) for the association of sleep hours, wake-up time, and bedtime, with breakfast skipping and having SMS meals. Results: Elementary schoolchildren sleeping for ≥9 hours were more likely to have parents/guardians in their 30s, who were full-time parents and maintained a healthy diet. Wake-up times and bedtimes were significantly associated with breakfast skipping frequency. Compared to the wake-up time from 6:30 to 6:59, ORs for the frequency of skipping breakfast was 0.52 (95% CI: 0.36–0.74, *p* < 0.001), for those waking before 6:29 and 2.23 (95% CI: 1.64–3.04; *p* < 0.001) for those waking after 7:00.Frequency of skipping breakfast decreased with earlier bedtimes. Compared to bedtimes from 21:00 to 21:59, ORs for having SMS meals were 0.64 for those with bedtimes before 21:00 (95% CI: 0.25–1.64) and 2.35 for those with bedtimes after 22:00 (95% CI: 1.77–3.11, *p* < 0.001). Compared to wake-up times from 6:30 to 6:59, waking up both before 6:29 (OR = 0.77, 95% CI: 0.63–0.94, *p* < 0.01) and at 7:00 or after (OR = 1.30, 95% CI: 1.00–1.69, *p* = 0.05) were associated with breakfast and dinner with SMS meals. A statistical interaction existed between wake-up time/bedtime and breakfast skipping. Compared to those waking before 6:30 and sleeping before 22:00, waking after 7:00 and sleeping after 22:00 was associated with skipping breakfast (OR = 2.11, 95% CI: 1.48–3.01; *p* for interaction = 0.04). Thus, children should sleep before 22:00 and wake up before 7:00 to prevent breakfast skipping and ensure a well-balanced diet with SMS meals. The ‘Early to Bed, Early to Rise, and Don’t Forget Your Breakfast’ initiative significantly improved elementary schoolchildren’s eating habits. Conclusions: Sleep hours, bedtime, and wake-up times are important factors affecting Japanese schoolchildren’s dietary habits, specifically in terms of breakfast skipping and having SMS meals.

## 1. Introduction

School years are an important period for children to learn eating habits that form the basis of lifelong health. Sleep is an essential resting activity for promoting and maintaining health. Breakfast contributes to the regulation of circadian rhythms, and it has been reported that skipping breakfast may lead to sleeping late and waking up late [1,2]. Therefore, ‘Food and Nutrition Education’ (called Shokuiku in Japan) is being implemented in elementary schools with the slogan ‘Early to Bed, Early to Rise, and Don’t Forget Your Breakfast’ [3,4,5]. Moreover, as children’s dietary habits are easily influenced by family lifestyles, it is necessary to learn about food education at both school and home [6,7].

There are also reports that associate eating patterns with sleep quality [8]. In February 2024, the ‘Sleep Guide for Health 2023’ [9] was revised for the first time in 10 years by the Ministry of Health, Labour and Welfare of Japan. This guide recommends that elementary schoolchildren should sleep for 9–12 h and receive early intervention, by ensuring that they go to bed early, wake up early [1,10], eat breakfast [2], exercise regularly, and reduce screen time [11]. However, this guide does not provide specific times for waking up or going to bed.

For behavioural change, it is not enough to know just the length of sleep needed; specific wake-up and sleep times are also necessary.

It has been suggested that ‘breakfast is the most important meal of the day and has a positive impact on children’s health and well-being,’ and review studies [12,13] on breakfast skipping among children and adolescents have reported a possible association with lower nutrient intake, academic performance, and dietary quality, as well as an increased prevalence of overweight and obesity.

It has been suggested that even when sufficient sleep time is secured, if the time of wake-up and going to bed are not appropriate, this may lead to the skipping of breakfast [14,15,16]. It has been reported that people who acquire healthy lifestyle habits as adults already developed good lifestyle habits during their school days [17].

The Fourth Basic Plan for the Promotion of ‘Shokuiku’ [18] promotes the practice of nutritionally balanced ‘Japanese style dietary habits,’ which consist of meals having the staple food, main dish, and side dish (SMS). It has been suggested that the ‘Dietary Reference Intakes for Japanese’ [19] can be met by consuming two or more SMS meals a day [20]. The sleep guide [9] also recommends building well-balanced dietary patterns, mainly for SMS meals, to ensure children receive the necessary nutrients.

However, only a few studies examined the relationship between a well-balanced meal—such as one including ‘staple food’, ‘main dish’, and ‘side dish’—and the wake-up time and bedtime [21], although several studies researched how wake-up times and bedtimes are associated with skipping breakfast [14,15,16]. Moreover, while there are studies on wake-up times and skipping breakfast, there has been little research examining the contents of breakfast [21]. Furthermore, to the best of our knowledge, no study has examined whether specific wake-up times and bedtimes (sleep hours) increase the frequency of SMS meals.

Saga Prefecture is implementing the 4th Saga Prefecture Food Education Promotion Plan [22] in line with the Fourth Basic Plan for the Promotion of ‘Shokuiku’ [18]. However, health issues are recognized across a wide range of age groups, from school children to adults. Saga Prefecture has the highest national rate of diabetes (HbA1c of 6.5% or higher) based on health screening results for people aged 40 to 74, and the rates of individuals with and at risk of metabolic syndrome are also higher compared to other prefectures [23]. Further, the percentage of 12-year-old boys and girls with high obesity index (indicators for school-age children in Japan) is 12.3%, higher than the national average of 11.5% [24]. 

Therefore, we conducted a cross-sectional study with children from seven elementary schools in Saga Prefecture and evaluated the relationship between sleep hours, wake-up time, and bedtime, while focusing on the skipping of breakfast and having Japanese-style eating habits (SMS meals).

## 2. Materials and Methods

### 2.1. Study Participants

A cross-sectional study was conducted from November 2015 to March 2016 with parents and guardians of children attending seven elementary schools (1st to 6th grades boys and girls aged between 6 and 12 years old) in Saga Prefecture, Japan.

In the case of children who had brothers or sisters in the same elementary school, there was high possibility that they have the same food and lifestyle habits. Therefore, to avoid duplicate answers, the study included only the first child; that is, the older brother or sister was given priority. We distributed 3332 questionnaires with the cooperation of each school and received 2457 responses (recovery rate: 73.7%).

### 2.2. Measurement

We investigated the children’s diet and lifestyle using three types of anonymous self-administered questionnaires: (A) ‘Children’s dietary habits’, (B) ‘Children’s lifestyle habits’ and (C) ‘health literacy of parents/guardians’. Of these, two questionnaires, (A) and (B), were used in this study. This study focused on everyday weekday lifestyle habits, regarding sleep and eating behaviours. Sleep duration was calculated from normal weekday wake-up and bedtimes. The qualitative dietary record was evaluated based on breakfast, dinner, and snacks for one typical weekday when school lunches are provided.

Questionnaire (A) focused on children’s weekday meals, and questionnaire (B) asked for basic information such as the age and job of respondents as well as children’s wake-up time, bedtime, breakfast skipping, television and game time (total screen time), physical activity time, and cavities.

Screen time per day was calculated by total weekly time based on the television and game time on weekdays and holidays, and this was classified into two groups (<120 and ≥120 min). Physical activity per day was calculated by total activity time per week and classified into three groups (0, <60, and ≥60 min). Dental caries was classified into two groups: ‘no history of dental caries’ consisting of schoolchildren who had never had dental caries and ‘with a history of dental caries’ consisting of schoolchildren who had experienced dental caries, including those who had already been treated.

Moreover, sleep hours were calculated from bedtime and wake-up time and classified into two groups (<9 or ≥9 h). Wake-up time was classified into three groups (before 6:29, from 6:30 to 6:59, and after 7:00). Bedtime was classified into three groups (before 20:59, from 21:00 to 21:59, and after 22:00).

### 2.3. Outcomes

Breakfast skipping was classified into two groups: breakfast skipping group (skipping more than once a week) and breakfast eating group (skipping less than once a week). Japanese dietary habits (SMS meals) were classified based on questionnaire (A).

Questionnaire A was a qualitative dietary record and contained questions about breakfast, dinner, and snacks on a typical weekday when school lunches were served and there were no special events. It included the names of dishes and foods, but did not include food weights, so that we could evaluate SMS meals.

The categorisation of SMS was determined based on the ‘Japanese Food Guide Spinning Top’ [25] prepared by the Ministry of Health, Labour and Welfare and the Ministry of Agriculture, Forestry and Fisheries: staple foods are grain-based dishes such as rice, miscellaneous cereals, bread, and noodles; main dishes are protein rich dishes, such as meats, fishes, soybeans, and eggs; and side dishes include vegetables, potatoes, seaweeds, and mushrooms. We defined ‘the group with SMS meals’ as those who had SMS meals for both breakfast and dinner. ‘Without SMS meal group’ did not have SMS meals for either breakfast or dinner.

### 2.4. Statistical Analysis

Participants’ characteristics were compared and examined based on children’s sleep hours, wake-up times, and bedtimes as well as their lifestyles and dietary habits using the chi-square test. The medians of physical activity were comparatively examined using the Wilcoxon rank-sum test. Logistic regression analyses were used to estimate odds ratios (ORs) and 95% confidence intervals (CIs) for the association of sleep hours, wake-up time, and bedtime, while considering breakfast skipping and having SMS meals.

For multivariate analysis, we adjusted for the respondents’ age and job as well as the children’s physical activity, screen time, and dental caries.

We conducted analyses stratified by (wake-up time, bedtime) to assess possible interactions with these factors. The cross-product terms of these factors (wake-up time, before 6:30, 6:30–6:59, 7:00 or after; bedtime, before 22:00; 22:00 or after) and 2 categories of breakfast skipping (yes/no) as well as SMS meals (with/without) were introduced into the model. The *p*-value for interaction was calculated by a likelihood ratio test comparing models with and without the interaction terms.

Data were analysed using SAS statistical package release ver. 9.4 and JMP pro ver. 16.2 (SAS Institute, Cary, NC, USA). Statistically significant differences were *p* < 0.05 for all analyses.

### 2.5. Ethical Considerations

This survey was conducted according to the ‘Ethical Guidelines on Epidemiological Studies’ established by the Japanese Government (Ministry of Health, Labour and Welfare and Ministry of Education, Culture, Sports, Science and Technology) [26]. The Ethics Committee of Tohoku University Graduate School of Medicine approved the research protocol (No. 2015–1–810, 2019–1–482). The survey was anonymous, and submission of the questionnaire was regarded as consent to participate.

## 3. Results

A total of 2457 survey participants responded to the questionnaire. Responses with missing data for breakfast skipping (n = 58), respondent age and work (n = 36), dental caries experience (n = 15), screen time (n = 20), and physical activity (n = 25) were excluded, resulting in a final survey sample with 2303 participants.

Table 1 presents the characteristics of participants. They shared that, overall, 1153 (50.1%) children slept less than 9 h. The most common wake-up time was 6:30 to 6:59, accounting for 1356 children (58.9%). The most common bedtime was at 21:00 to 22:00, accounting for 1130 children (49.1%).

More than 95% respondents were aged 30 to 49 years (30s: 46.9%; 40s: 48.4%). Children who slept for fewer hours (<9 h) than those who slept longer (≥9 h) were more likely to have parents/guardians in their 40s (vs. 30s; *p* < 0.001) that are full-time employees (vs. full-time parents; *p* < 0.001). They were more likely to engage in little or no physical activity (*p* < 0.001), have greater screen time (*p* < 0.001), have with a history of dental caries (*p* < 0.001), and skip breakfast more often (*p* < 0.001; see Table 1).

Compared to children with later wake-up times (after 7:00), those with earlier wake-up times (before 7:00) tended to have more parents/guardians in their 30s (vs. 40s; *p =* 0.01) and full-time parents (vs. full-time employees; *p* = 0.03); they have lower screen time (*p* < 0.001), skip fewer breakfasts (*p* < 0.001), and have more SMS meals (*p* < 0.001; Table 1).

Compared to children with later bedtimes (after 22:00), those with early bedtimes (before 22:00) were more likely to have parents/guardians in their 30s (vs. 40s; *p* < 0.001) that are full-time parents (vs. full-time employees; *p* < 0.001); they have more physical activity (*p* < 0.001), lower screen time (*p* < 0.001), and less experience with dental caries (*p* < 0.001). They also skip fewer breakfasts (*p* < 0.001) and have more SMS meals (*p =* 0.01; Table 1).

Table 2 presents results of the multivariate analysis of sleep hours, wake-up times, and bedtimes with breakfast skipping as well as SMS meals. Compared with those who sleep for longer periods (≥9 h), those with fewer hours of sleep (<9 h) had a 35% higher chance of skipping breakfast; the multivariate adjusted OR was 1.35 (95%CI: 1.03–1.76).

Children with earlier wake-up times (before 6:29) did not skip breakfast, and the multivariate adjusted OR was 0.52 (95%CI: 0.36–0.74); furthermore, those with late wake-up times (after 7:00) tended to skip breakfast, and their multivariate adjusted OR was 2.23 (95%CI: 1.64–3.04), when compared with those with wake-up times between 6:30 and 6:59. Compared with those whose bedtimes ranged from 21:00 to 21:59, those with early bedtimes (before 8:59) did not skip breakfast (multivariate adjusted OR: 0.64, 95%CI: 0.25–1.64), and those with late bedtimes (after 22:00) skipped breakfast (multivariate adjusted OR: 2.35, 95%CI: 1.77–3.11). Furthermore, those with earlier wake-up times (before 6:29) have SMS meals, with a multivariate adjusted OR of 0.77 (95%CI: 0.63–0.94), and those with late wake-up times (after 7:00) tended to not have SMS meals, with the multivariate adjusted OR of 1.30 (95%CI: 1.00–1.69), when compared with those with wake-up times between 6:30 and 6:59 (Table 2).

We also evaluated whether the observed associations of wake-up time with breakfast skipping were modified by bedtime (before 22:00/22:00 or after; Table 3). Compared with those with wake-up times before 6:30 and bedtimes before 22:00, the multivariate OR for those with wake-up times of 7:00 or after and bedtimes of 22:00 or after, with breakfast skipping, was 9.10 (95%CI: 5.11–16.20; *p* for interaction = 0.25).

We evaluated whether the observed associations of wake-up times with having SMS meals were modified by bedtime (before 22:00/22:00 or after; Table 4). Compared with those with wake-up times before 6:30 and bedtimes before 22:00, the multivariate OR for those with wake-up times of 7:00 or after and bedtimes of 22:00 or after, with having SMS meals, was 2.11 (95%CI: 1.48–3.01; *p* for interaction = 0.04).

## 4. Discussion

We examined the relationship of children’s sleep time, wake-up time, and bedtime, while considering breakfast skipping and SMS meals based on a cross-sectional survey of about 2000 primary schoolchildren and parents/guardians that covered 70% of households in the area with one or more school-age children. Our results show the inadequacy of children’s sleep duration. Half of the children did not sleep for 9–12 h as recommended by the sleep guide [9]. In previous Japanese studies, the average sleep duration was 8 h 35 min for first-grade children, 7 h 55 min for sixth-year children [27], 8 h 45 min for 10–14-year olds [28], and 8 h 54 min for 6–12-year olds [29]; these durations are similar to our results.

Among those aged ≥20 years, 60.9% satisfied the recommended sleep duration of ≥6 h for adults [30]. The Organization for Economic Co-operation and Development [31] reported that the average amount of sleep in 33 countries was 8 h 28 min. Japan has an average sleep time of 7 h 22 min, which is the shortest out of 33 countries. It has been reported that short sleep durations increase the risks of the onset of various diseases and mortality [32], such as due to obesity [33,34,35], heart disease [36], lifestyle-related diseases [37], and dementia [38]. Compensating for lack of sleep during weekdays by oversleeping on weekends can cause social jet lag between weekdays and weekends [39]. Such social jet lag might cause chronic sleep deprivation and misalignment of the circadian rhythm, leading to increased risk of obesity, heart disease, and depression [40].

The rate of skipping breakfast was 12.9% in our study, and 11.0% [41], 4.0% [42], 6.3% [7], and 21.2% [43] in previous studies. Similarly to previous studies, our results suggest a significant association of short sleep hours, late wake-up times, and late bedtimes with breakfast skipping [44]. Moreover, studies with those in their 20s–30s have shown an association between late bedtimes and breakfast skipping [45]. Previous studies [17] also reported that those who eat regular meals during school age tend to have breakfast in adolescence, so the slogan ‘Early to Bed, Early to Rise, and Don’t Forget Your Breakfast’ is a reference to a way of attaining desirable lifestyle habits [3,4].

While it is important to address breakfast skipping from school age, a survey of 11-year olds in Saga City, Saga Prefecture, in 2017 [46] showed that 16.8% skipped breakfast, an increase from the 12.9% reported in 2016. Another survey in Saga Prefecture targeting adults in 2020 [47] found that 11.6% of men and 8.5% of women habitually skipped breakfast, with 24.3% of men and 16.4% of women reporting that they began the habit of breakfast skipping between primary and high school. In this study, 58.1% of schoolchildren in 2016 had two or more servings of SMS meals per day, while a 2017 study of schoolchildren reported 29.1% had SMS at breakfast [46]. In 2020, less than half of the adults had two or more servings of SMS per day, and about 70% of respondents reported difficulty in including side dishes in their children’s meals [47].

The Fourth Saga Prefecture Nutrition Education Promotion Plan (implemented in 2021) [22] aims for over 60% of the population to have a balanced diet with a meal pattern of SMS at least twice a day as well as reduce breakfast skipping to less than 9.7% for men and 8.0% for women by 2025. As part of specific initiatives, families, schools, communities, companies, and private organisations are collaborating to promote the ‘Early to Bed, Early to Rise, and Don’t Forget Your Breakfast’ initiative and encourage individuals and families to strive for a balanced diet combining SMS. Furthermore, the Fourth Basic Plan for Food Education Promotion mentions the reduction in citizens’ breakfast skipping, aiming to reduce the percentage of children who do not eat breakfast at all or eat very little from 4.6% (in 2019) to 0% (by 2025) [18].

Eating meals with SMS twice or more a day makes it easier to meet dietary reference intakes [20], but since few people manage to have meals with SMS, it is considered necessary to propose meals that include SMS. For nutritional improvement, waking up before 6:30 and going to bed before 9:30 to ensure 9 h of sleep is recommended.

This study further observed a tendency for children with short sleep durations and late bedtimes to have no physical activity. Previous studies also suggested an association between good sleep habits and physical activity [41]. We found that those with late wake-up times and late bedtimes tended to have longer screen time, consistent with previous studies suggesting that late bedtimes are associated with longer screen times [48,49]. A recent study of Japanese schoolchildren found that ‘longer screen time tends to increase energy intake, but also decrease intake of nutrients such as protein, minerals, vitamins, and dietary fibre’ [50]. Similarly to our study, the presence of dental caries has also been reported to be associated with short sleep duration [51]. Therefore, in this study, we also took into account total screen time and the presence or absence of dental caries through statistical adjustments.

The strength of this study is that it collected data from over 2000 children. However, it has limitations. First, because we used a self-administered questionnaire completed by parents/guardians, there is a possibility of underreporting or overreporting. In particular, evaluations of qualitative dietary records by people other than the person themselves may be more likely to deviate from the true values. However, the SMS meal, which is the dietary outcome indicator in this survey, is a qualitative evaluation rather than a quantitative evaluation.

Second, qualitative dietary records were for one day, making it difficult to grasp long-term eating habits in a short period. However, compared to the National Health and Nutrition Survey in 2019 (ages 7–14 years: 454 individuals), this study, with over 2000 data points, can be considered to at least estimate the values for schoolchildren in the survey area.

Third, the sample size for specific outcomes was not calculated in advance, which is a limitation of this study. However, the purpose of this study is to understand the actual situation of the various living environments and health issues of children in the area, and it is a survey research aimed at conducting a complete survey of the area.

Fourth, one of the limitations of this study is that weekend data was not taken into account, while the main focus of this study is to propose specific behavioural changes on weekdays.

Fifth, this study focused on schoolchildren in a limited area of Japan, Saga Prefecture, and it may be difficult to generalise to the Japanese population as a whole, which is one of the limitations of this study.

Sixth, to protect personal information, it was not possible to obtain information of individual details regarding on the ages and genders/sex of the schoolchildren as well as responder, only age categories for responders (parents/guardians) were collected. While analysis linked to personal factors such as age and gender/sex has its limitations, this data provides highly valuable insights into the situation and trends among schoolchildren.

Notably, in cases where schoolchildren have siblings, data overlap occurs in the analysis concerning any factors. Therefore, the survey data consisted only of responses from the oldest brother or sister, who are likely to have the same eating patterns and lifestyle habits as their younger siblings, and efforts were made to improve the accuracy of data analysis.

Our results suggest the need for behavioural change among elementary schoolchildren in Saga Prefecture.

In this cross-sectional study, we observed that waking up after 7:00 and going to bed after 22:00 at the latest and obtaining less than 9 h of sleep were associated with skipping breakfast. A statistically significant association was also observed between ‘waking up by 7:00,’ and ‘having SMS meals.’ In light of these results, it is advisable for schoolchildren to wake up by 7:00 at the latest, and to go to bed by latest 22:00.

Behavioural change is difficult and requires specific information. Our findings may encourage behavioural change by providing specific recommendations for wake-up and bedtime times, leading to ‘daily recommended eating habits’ including breakfast and SMS meals.

## 5. Conclusions

This cross-sectional study reported that approximately half of the schoolchildren surveyed were not achieving the nine hours of sleep recommended by the ‘Sleep Guide for Health 2023’ [9], revealing health issues facing Japanese schoolchildren. Respondent’s attributes such as their age and occupation were related to children’s sleep duration, wake-up time, and bedtime. Our results provide specific times to guide behavioural change, rather than just encouraging messages like ‘Early to Bed, Early to Rise, and Don’t Forget Your Breakfast.’ Further research, including studies focusing on parents/guardians, is needed to address sleep-related health issues among Japanese schoolchildren.

## Figures and Tables

**Table 1:** Characteristics of the study participants (respondents and children).

	Overall		Sleep Hours					Wake-Up Time							Bed Time						
	n = 2303		<9 h n = 1153 (50.1%)		≥9 h n = 1150 (49.9%)		*p*	Before 6:29 n = 621 (27.0%)		6:30–6:59 n = 1356 (58.9%)		After 7:00 n = 326 (14.2%)		*p*	Before 20:59 n = 116 (5.0%)		21:00–21:59 n = 1130 (49.1%)		After 22:00 n = 1057 (45.9%)		*p*
	n	%	n	%	n	%		n	%	n	%	n	%		n	%	n	%	n	%	
**Respondent’s age**																					
20s or younger	48	2.1	18	1.6	30	2.6	<0.001	9	1.5	25	1.8	14	4.3	0.01	2	1.7	24	2.1	22	2.1	<0.001
30s	1081	46.9	464	40.4	617	53.7		298	48.0	653	48.2	130	39.9		84	72.4	590	52.2	407	38.5	
40s	1114	48.4	631	54.7	483	42.0		295	47.5	649	47.9	170	52.2		28	24.4	498	44.1	588	55.6	
50s and older	60	2.6	40	3.5	20	1.7		19	3.1	29	2.1	12	3.7		2	1.7	18	1.6	40	3.8	
**Respondent’s job**																					
Full-time employee	518	22.5	290	25.2	228	19.8	<0.001	128	20.6	313	23.1	77	23.6	0.03	8	6.9	247	21.9	263	24.9	<0.001
Part-time or temporary	1059	46.0	552	47.9	507	44.1		277	44.6	610	45.0	172	52.8		53	45.7	474	42.0	532	50.3	
Self-employed or helping	97	4.2	52	4.5	45	3.9		26	4.2	54	4.0	17	5.2		4	3.5	49	4.3	44	4.2	
Full-time parent	550	23.9	221	19.2	329	28.6		167	26.9	331	24.4	52	16.0		46	39.7	317	28.1	187	17.7	
During leave or childcare leave	29	1.3	11	1.0	18	1.6		10	1.6	16	1.2	3	0.9		2	1.7	18	1.6	9	0.9	
Unemployed	8	0.4	4	0.4	4	0.4		4	0.6	3	0.2	1	0.3		0	0.0	4	0.4	4	0.4	
Other	42	1.8	23	2.0	19	1.7		9	1.5	29	2.1	4	1.2		3	2.6	21	1.9	18	1.7	
**Physical activity**, minute/day																					
Median	49.29		47.14		51.43		0.43 ^a^	47.14		51.43		51.43		0.27 ^a^	46.43		51.43		47.14		0.41 ^a^
IQR (25%ile–75%ile)	(17.14–85.71)		(17.14–92.50)		(21.43–81.43)			(17.14–80.71)		(19.29–88.93)		(17.14–85.71)			(20.18–80.36)		(24.11–81.43)		(17.14–94.29)		
0 min/day	219	9.5	135	11.7	84	7.3	<0.001	63	10.1	115	8.5	41	12.6	0.18	6	5.2	82	7.3	131	12.4	<0.001
<60 min/day	1118	48.6	529	45.9	589	51.2		305	49.1	656	48.4	157	48.2		63	54.3	572	50.6	483	45.7	
≥60 min/day	966	42.0	489	42.4	477	41.5		253	40.7	585	43.1	128	43.1		47	40.5	476	42.1	443	41.9	
**Screen time**, minute/day																					
Median	132.86		137.14		120.00		<0.001 ^a^	120.00		128.57		154.29		<0.001 ^a^	94.29		115.71		150.00		<0.001 ^a^
IQR (25%ile–75%ile)	(88.57–181.43)		(94.29–197.14)		(77.14–171.43)			(82.86–180.00)		(86.07–175.71)		(106.43–214.29)			(60.00–154.29)		(77.14–162.86)		(102.86–205.71)		
<120 min/day	1015	44.1	448	38.9	567	49.3	<0.001	304	49.0	614	45.3	97	29.8	<0.001	72	62.1	588	52.0	355	33.6	<0.001
≥120 min/day	1288	55.9	705	61.1	583	50.7		317	51.1	742	54.7	229	70.3		44	37.9	542	48.0	702	66.4	
**History of dental caries**																					
No	695	30.2	308	26.7	387	33.7	<0.001	204	32.9	400	29.5	91	27.9	0.20	50	43.1	386	34.2	259	24.5	<0.001
With	1608	69.8	845	73.3	763	66.3		417	67.2	956	70.5	235	72.1		66	56.9	744	65.9	798	75.5	
**Breakfast skipping**																					
Yes (skipping ≥1 time/week)	296	12.9	181	15.7	115	10.0	<0.001	43	6.9	164	12.1	89	27.3	<0.001	5	4.3	86	7.6	205	19.4	<0.001
No	2007	87.2	972	84.3	1035	90.0		578	93.1	1192	87.9	237	72.7		111	95.7	1044	92.4	852	80.6	
**SMS meals for breakfast and dinner (twice/day)**																					
Without	1338	58.1	663	57.5	675	58.7	0.56	324	52.2	796	58.7	218	66.9	<0.001	57	49.1	637	56.7	644	60.9	0.01
With	965	41.9	490	42.5	475	41.3		297	47.8	560	41.3	108	33.1		59	50.9	493	43.6	413	39.1	

χ2 test/^a^: Wilcoxon rank-sum test/Kruskal–Wallis test; SMS: staple foods, main dishes, and side dishes. IQR: interquartile range.

**Table 2:** Results of multivariate analysis of sleep hours, wake-up time, and bedtime with breakfast skipping as well as SMS meals.

		Breakfast Skipping			SMS Meals (2 Times/Day)		
	N	Yes/No	Crude OR (95% CI)	Model 1 OR (95% CI)	Without/With	Crude OR (95% CI)	Model 1 OR (95% CI)
**Sleep hours**							
<9 h	1153	181/972	1.68 (1.31–2.15)	1.35 (1.03–1.76)	663/490	0.95 (0.81–1.12)	0.89 (0.74–1.06)
≥9 h	1150	115/1035	1.00 (ref)	1.00 (ref)	675/475	1.00 (ref)	1.00 (ref)
**Wake** **-up time**							
Before 6:29	621	43/578	0.54 (0.38–0.77)	0.52 (0.36–0.74)	324/297	0.77 (0.63–0.93)	0.77 (0.63–0.94)
6:30–6:59	1356	164/1192	1.00 (ref)	1.00 (ref)	796/560	1.00 (ref)	1.00 (ref)
7:00 or after	326	89/237	2.73 (2.04–3.66)	2.23 (1.64–3.04)	218/108	1.42 (1.10–1.83)	1.30 (1.00–1.69)
**Bedtime**							
Before 20:59	116	5/111	0.55 (0.22–1.38)	0.64 (0.25–1.64)	57/59	0.75 (0.51–1.10)	0.76 (0.51–1.13)
21:00–21:59	1130	86/1044	1.00 (ref)	1.00 (ref)	637/493	1.00 (ref)	1.00 (ref)
22:00 or after	1057	205/852	2.92 (2.24–3.82)	2.35 (1.77–3.11)	644/413	1.21 (1.02–1.43)	1.11 (0.92–1.33)

Model 1: Multivariate-adjusted for age (20s or younger, 30s, 40s, 50s, or older), job (full-time employee, part-time or temporary, self-employed or helping, full-time parent or other), screen time at video games and television (minutes/day: <60, 60–119, 120–179, 180–239, 240–299, and ≥300), physical activity (minutes: 0, <60, and ≥60), and history of dental caries (no and with). OR: odds ratio; CI: confidence interval; SMS: staple foods, main dishes, and side dishes.

**Table 3:** Multivariate odds ratio for the association of wake-up time, bedtime with breakfast skipping.

Wake-Up Time	Before 6:30		6:30–6:59		7:00 or After		*p* for Interaction
Bedtime	Skipping (Yes/No)	Multivariate Adjusted OR (95% CI)	Skipping (Yes/No)	Multivariate Adjusted OR (95% CI)	Skipping (Yes/No)	Multivariate Adjusted OR (95% CI)	
Before 22:00	17/415	1.00 (ref)	61/656	2.32 (1.32–4.05)	13/84	3.22 (1.48–7.00)	0.25
22:00 or after	26/163	2.76 (1.43–5.33)	103/536	3.96 (2.30–6.79)	76/153	9.10 (5.11–16.20)	

Multivariate-adjusted for age (20s or younger, 30s, 40s, and 50s or older), job (full-time employee, part-time or temporary, self-employed or helping, and full-time parent or other), screen time at video games and television (minutes/day: <60, 60–119, 120–179, 180–239, 240–299, and ≥300), physical activity (minutes: 0, <60, and ≥60), and history of dental caries (no and with). OR: odds ratio; CI: confidence interval.

**Table 4:** Multivariate odds ratio for the association of wake-up time, bedtime with SMS meals.

Wake-Up Time	Before 6:30		6:30–6:59		7:00 or After		*p* for Interaction
Bedtime	SMS (2 Times/Day) Without/With	Multivariate Adjusted OR (95% CI)	SMS (2 Times/Day) Without/With	Multivariate Adjusted OR (95% CI)	SMS (2 Times/Day) Without/With	Multivariate Adjusted OR (95% CI)	
Before 22:00	210/222	1.00 (ref)	426/291	1.52 (1.19–1.94)	58/39	1.43 (0.90–2.26)	0.04
22:00 or after	114/75	1.39 (0.97–2.00)	370/269	1.35 (1.04–1.74)	160/69	2.11 (1.48–3.01)	

Multivariate-adjusted for age (20s or younger, 30s, 40s, and 50s or older), job (full-time employee, part-time or temporary, self-employed or helping, and full-time parent or other), screen time at video games and television (minutes/day: <60, 60–119, 120–179, 180–239, 240–299, and ≥300), physical activity (minutes: 0, <60, and ≥60), and history of dental caries (no and with). SMS: staple foods, main dishes, and side dishes; OR: odds ratio; CI: confidence interval.

## Data Availability

The original contributions presented in this study are included in the article. Further inquiries can be directed to the corresponding author.

## Abbreviations

The following abbreviations are used in this manuscript:

CI	Confidence interval
OR	Odds ratio
SMS meal	Meal of staple food, main dish, and side dish
IQR	Interquartile range
